# Early pain experiences in dairy calves on pain sensitivity later in life

**DOI:** 10.3168/jdsc.2023-0420

**Published:** 2023-11-17

**Authors:** Zimbábwe Osório-Santos, Thomas Ede, Maria José Hötzel, Daniel M. Weary, Marina A.G. von Keyserlingk

**Affiliations:** 1 Animal Welfare Program, Faculty of Land and Food Systems, The University of British Columbia, Vancouver, BC, V6T 1Z6, Canada; 2 Laboratório de Etologia Aplicada e Bem-Estar Animal, Universidade Federal de Santa Catarina, Florianópolis 88034-001, SC, Brazil

## Abstract

In humans, early painful experiences can increase pain sensitivity later in life, but little is known regarding this phenomenon in cattle. This study assessed if a painful event early in life affects later pain sensitivity in 40-d-old calves. Holstein calves (n = 26) were randomly assigned to control or treatment conditions, blocked within sex and birth weight. At 9.5 ± 1.8 d old, treatment calves had 1 horn bud disbudded using caustic paste, whereas control calves had 1 horn sham disbudded; in both cases, multimodal pain control was provided. All calves had the contralateral horn bud disbudded using a hot iron 4 wk later, again with multimodal pain control. Mechanical nociceptive responses were assessed weekly using an algometer applied adjacent to both horn buds and on the rump, beginning 3 d before the first disbudding and ending 30 d after the second disbudding. Following the second disbudding, both groups of calves showed evidence of increased sensitivity (i.e., algometer pressure declined 3.69 ± 0.60 √N to 2.13 ± 0.70 √N) on the contralateral bud, but there was no difference between the control group and the treatment groups. An interaction between treatment and time, likely driven by treatment differences, was found on the rump when tested 5 h after the second disbudding event. These responses are not consistent with the hypothesis that an early pain experience results in increased sensitivity to later painful experiences.

## Highlights

• Early painful experiences may alter general nociceptive responses later in life in calves.
• Our findings do not support our hypothesis of hyperalgesia due to early painful experiences.
• Calves vary in terms of their nociceptive responses.

## Graphical Abstract

**Figure:**
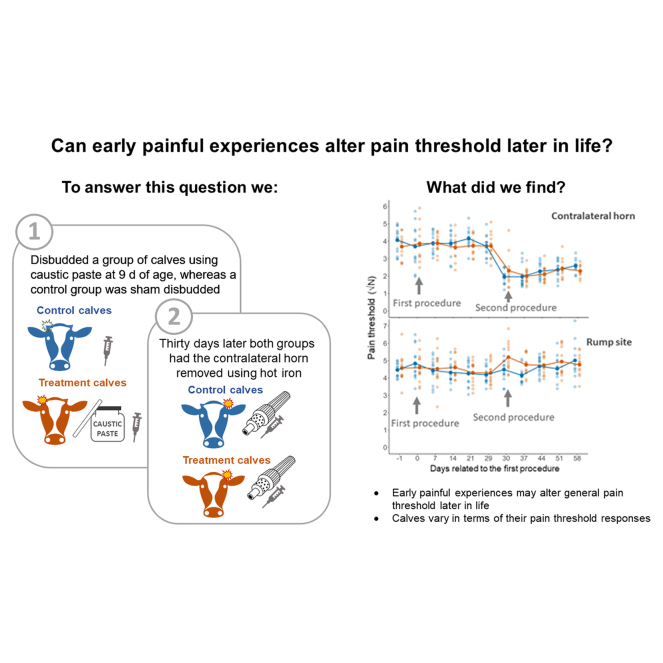
**Summary** Painful experiences early in life can affect pain sensitivity later in life in humans, but this phenomenon has seldom been studied in animals. We tested the effect of an early pain experience on later pain sensitivity in calves by assessing responses to disbudding 1 horn bud at 40 days of age for control calves that had not experienced a previous disbudding event versus treatment calves that had their other horn bud disbudded 30 days earlier. Pain sensitivity was tested in the rump, adjacent to the site of the recent horn bud injury, and at the other horn bud site (i.e., treatment calves had been injured 30 days earlier). Contrary to our expectation, calves that had previously experienced a painful event tended to be less sensitive to pain than control calves.

Disbudding is a routine management practice on dairy farms (USDA-NAHMS, 2014), typically performed in the first month of life (Shivley et al., 2019). Early painful experiences can result in changes in the nervous system, such as increased sensitivity of the dorsal horn nociceptive circuits and altered pattern of the descending pain control system (Schwaller and Fitzgerald, 2014). These disruptions help explain pain threshold shifts in both humans (Beggs et al., 2012) and rodents (Williams and Lascelles, 2020). Specifically, painful experiences in early life increase pain sensitivity (i.e., decreasing pain thresholds), an effect that can persist for months or years (Taddio et al., 1997; Brummelte et al., 2015).

Previous studies on farm animals have explored the effect of early pain on general sensorial sensitivity (Clark et al., 2014; Adcock and Tucker, 2018) and behavioral responses (McCracken et al., 2010; Clark et al., 2014; Adcock and Tucker, 2020). Early painful experiences can cause a reduction in the overall pain threshold (Adcock and Tucker, 2018; Mirra et al., 2018) and an increased behavioral response following a second painful procedure in heifers (Adcock and Tucker, 2020) and lambs (McCracken et al., 2010). To our knowledge, the effects of early painful experiences on pain thresholds following later painful experiences in calves have not been explored. We assessed if an early painful experience, originating from the disbudding of 1 horn bud, would affect calf responses to the disbudding of the second horn bud 4 wk later. We predicted that animals who experienced early disbudding would show a more pronounced response to the subsequent disbudding event.

This study took place at The University of British Columbia (**UBC**) Dairy Education and Research Centre (Agassiz, BC, Canada) between September 2021 and February 2022. The project was approved by UBC's Animal Care Committee (#A16–0310).

Because no prior studies, to our knowledge, have examined the impact of prior painful experiences on nociceptive thresholds in dairy calves following a second painful experience, we did not undertake a formal power analysis. Instead, our sample size was determined based on earlier research that employed an algometer to assess pain thresholds in calves following disbudding, in the rump (Adcock and Tucker, 2018; 12 calves/treatment) and the horn bud (Mirra et al., 2018; n = 10–12 calves/treatment); based on these previous studies we opted for a sample size of 26 Holstein calves (13 calves/treatment). We enrolled 20 female and 6 males into the study (mean ± SD; BW = 40.21 ± 5.55 kg) that were kept in individual pens measuring 1 × 1.5 m, bedded with fresh sawdust for the first 5 d of life. On d 4, all calves were fitted with a single ear tag in each ear using an ear tagging device that punctured the ear (Allflex, Merck and Co. Inc., Rahway, NJ). Calves were then moved to a 35-m^2^ group pen on d 5 and housed in groups of 10. Fresh sawdust was added weekly. Calves had access to 12 L/d of pasteurized whole milk using automated feeders (CF 1000 CS Combi; DeLaval Inc., Tumba, Sweden) equipped with 1 teat and a raceway (0.4 × 1.5 m) that restricted access to a single calf at a time. Milk allowances accumulated at a rate of 5% of the daily allowance per hour from 2400 to 2000 h. The milk feeder delivered a minimum of 0.5 L and a maximum of 9.5 L per visit. Approximately 0.5 m adjacent to the milk feeder was a starter feeder (CF 1000 feeder, DeLaval Inc.), also equipped with a barrier (0.4 × 1.0 m) to allow access to a single calf at a time. All calves had ad libitum access to starter (20% CP texturized and consisting of 31.2% flake barley, 15.3% canola meal, 15.0% flaked corn, 12.3% soybean meal, 8.7% wheat, and 6.5% molasses; Richie Smith Feeds Inc., Abbotsford, BC, Canada). Calves were also provided ad libitum access to water and hay via automated Insentec feeders (RIC; Insentec B.V., Hokofarm Group, Emmeloord, the Netherlands).

The farm's calving schedule resulted in calves being gradually enrolled into the study from September 9 to December 18, 2021. Once enrolled, calves were paired with another calf of similar birth weight and having no more than a 1-d difference in birth date. Once the pair was formed, a randomization process was conducted using a website (https://www.random.org/) to assign one of the paired calves to the treatment group and its counterpart to the control group. Pairs were housed in the same pen, and randomization was done within blocks: sex (control: 3 males, 10 females; treatment: 3 males, 10 females) and birth weight (control: 40.44 ± 5.73 kg; treatment: 39.98 ± 5.64 kg). Each group underwent 2 procedures. At 9.5 ± 1.8 d old, treatment calves had one horn bud disbudded using caustic paste and control calves underwent a sham procedure (both interventions are described later, and henceforth referred to as the first procedure). Right and left horn buds were balanced across sex and treatment. Calves from both groups had the contralateral horn bud disbudded by hot iron 30 d later (referred to as the second procedure).

During the first procedure, 1 calf from each treatment was gently moved to a separate pen (2.0 m × 2.0 m), and both were sedated using a subcutaneous injection of xylazine (0.2 mg/kg, Rompun 20 mg/mL, Bayer, Leverkusen, Germany). After sedation, 1 horn bud was randomly selected for disbudding. Afterward, an anesthetic block (5 mL of 2% lidocaine, 1:100,000 epinephrine, Lido-2, Rafter8, Calgary, AB, Canada) was applied between the lateral *canthus* and the horn bud. After waiting 10 min for the anesthetic to take effect, the region around the horn bud was shaved and desensitization of the cornual nerve was confirmed by the absence of reaction to a needle prick immediately adjacent to where the caustic paste would be applied. During the first disbudding event where the calf exhibited a response during the needle prick test, such as ear flicking or attempting to lift its head, the test was repeated after a 3-min interval. None of the calves displayed a reaction during the second prick test. Caustic paste (calcium hydroxide 24.9%, sodium hydroxide 21.5%, Dr. Naylor, Morris, NY) was then applied to the horn bud area (~17 ± 2.2 mm in diameter) of the treatment calves; the amount of paste was estimated to be 0.30 ± 0.10 g/bud based upon pretreatment training using 6 nonexperimental calves. A ring of petroleum jelly (Original Vaseline, Unilever, Toronto, ON, Canada) was applied around the paste area to prevent spread of the caustic paste. Control calves were sham disbudded, which included all aspects, including the needle pricks, described above with the exception of the caustic paste application. Immediately following the application of the petroleum jelly, all calves received a subcutaneous injection of a nonsteroidal anti-inflammatory drug (0.5 mg/kg meloxicam; Metacam, 20 mg/mL, Boehringer Ingelheim International, Ingelheim, Germany). Following the first procedure, calves were placed in sternal recumbency and left to recover for 5 h before being returned to the home pen.

Calves from both groups were subjected to the second procedure (i.e., disbudding of the contralateral horn bud) 30 d later. The disbudding procedure was identical to that described previously, but instead of using caustic paste the horn bud was disbudded with a preheated hot iron (X30 1/2” Tip, Rhinehart, Spencerville, IN) that was ~500°C. However, one calf from the treatment group reacted after the corneal block was administered during the first and second needle prick tests; thus, an additional 2.5-mL lidocaine anesthetic block was administered. After a 5-min waiting period, there was no reaction to the needle prick and the disbudding procedure was performed. The hot iron was applied using minimal pressure, rotated gently back and forth for ~15 s, until a homogeneous copper-colored ring was formed around the bud; the horn bud was not removed after the heat cauterization. During the 5 h recovery period after each procedure, pain behaviors (see Faulkner and Weary, 2000; Winder et al., 2017) were recorded from video: (1) head rub, defined as head in contact with and moving against either a hind leg or against a wall of the pen; (2) ear flicks, defined as ears moving at least once back and forth in rapid succession; and (3) head shake, defined as the head moving rapidly from side to side at least once. Behaviors were scored as either 0, when not observed, or the specific frequency when observed. Two trained observers, 1 of whom was blind to treatment, scored videos continuously for 1 min every 5 min. The sums of all pain behaviors (no./min) from the 2 observers were correlated (r_243_ = 0.95, *P* < 0.001).

Mechanical nociceptive threshold (**MNT**) was assessed using an algometer with a 1-cm rubber tip (Wagner Force, One FDIX, Wagner Instruments, Greenwich, CT). The data were transformed from the kilogram-force unit (kgf) to newtons (N) by multiplying by 9.80665. Just before assessment, calves were blindfolded and gently restrained to reduce variation introduced by calf movements during measurement (Frahm et al., 2020). The MNT measurements were taken at d −3, −2, −1 before the first procedure, 5 h after and then on d 7, 8, 14, 15, 21, 22, 28, and 29 following the first procedure. On d 30, 1 measurement was taken 5 h after the second procedure, and on d 38, 39, 45, 46, 52, 53, 59, and 60, relative to the date of the first procedure.

The MNT of the horn bud was tested first laterally, second superior from the bud, and last at the point between the sacral vertebrae and the femur joint on both sides of the rump. The order of the left or right sides horn buds and rump measurements were balanced between treatments and sex. For each measure a hand was lightly placed on the region to be assessed and kept there until the calf stopped moving. The hand was then replaced with the algometer rubber tip. The holder then applied pressure at a constant intensity of ~1 kg/s until the animal moved its head away (MNT in the horn bud area) or lifted 1 of its legs (MNT at the rump). Due to the visibility of the wound, it was not possible to blind the operator of the algometer to treatment groups.

Calves were treated as the experimental unit in all analyses. The final sample included 13 calves/treatment. All analyses were performed using R (R Core Team, 2021). To detect and examine outliers we used a mixed approach: (1) each experimental day was screened graphically, with the intention of identifying any between-animal outliers. Using the interquartile range method (Yang et al., 2019) 24 outliers were identified; (2) using a within-animal approach, where data from each animal was divided into 3 different time periods: baseline (d −3, −2, −1), between the first and second procedure (d 7, 8, 14, 15, 21, 22, 28, 29), and after the second procedure (d 38, 39, 45, 46, 52, 53, 59, and 60). Mean and standard deviation were generated for each of these 3 time periods, and the outliers identified using the first approach were assessed using z-scores. Because no data point surpassed the 2.5 standard deviation threshold, no outliers were removed from the final analysis.

Considering that we predicted that treatment calves would show a lower nociceptive threshold than control calves following the second procedure, we opted to build only 1 model for each body region, comparing the measurements taken in the days before the second procedure and the measurement taken 5 h after. A mixed model (function lmer, package lme4) was used to explore differences between and within treatment groups over time. Time, treatment, and their interactions were set as fixed effects, animal was set as a random effect, and time was treated as a continuous variable. Normality and homoscedasticity of residuals were checked graphically, and data were square-root transformed to improve model fit. We included all significant interactions (*P* < 0.05) in the final model. Measurements taken at the lateral and superior sites were averaged across the first and second procedures, and across the left and right sides of the rump. Results are presented in the square-root-transformed scale.

Because calves exhibited a very low frequency of some pain behaviors, we aggregated the 3 pain behaviors (ear flicks, head rubs, and head shakes) as described in previous articles on pain from disbudding (Winder et al., 2017; Ede et al., 2020). A simple *t*-test was used to explore possible differences between groups in terms of the aggregated pain behaviors exhibited in the 5 h following the first procedure, and again following the second procedure; results are presented as means ± standard error.

As expected, only treatment calves showed a decline in MNT at the first procedure site after receiving the intervention, and MNT values remained lower in the subsequent weeks (Figure 1). Given this initial painful experience, we had predicted that these calves would show lower MNT than control calves following their second procedure at the site of the second procedure, as well as at the site of the rump. However, none of our results were consistent with these predictions. Treatment calves showed lower nociceptive pain thresholds at the first procedure site after receiving the second procedure in the contralateral horn (treatment − control = −0.54 ± 0.24; *t*_1, 24_ = −2.20, *P* = 0.03; Figure 1A). We found no evidence of a treatment difference in the decline in MNT at the site of the second procedure (treatment − control = 0.09 ± 0.28; *t*_1, 50_ = 0.32; *P* = 0.75; Figure 1B). For the rump, we noted an interaction between treatment and time (interaction estimate = 0.54 ± 0.27; *t*_1, 24_ = 2.03, *P* = 0.05; Figure 1C) driven by treatment calves showing elevated rump MNT measures over the first 2 d after the second procedure.

**Figure 1:**
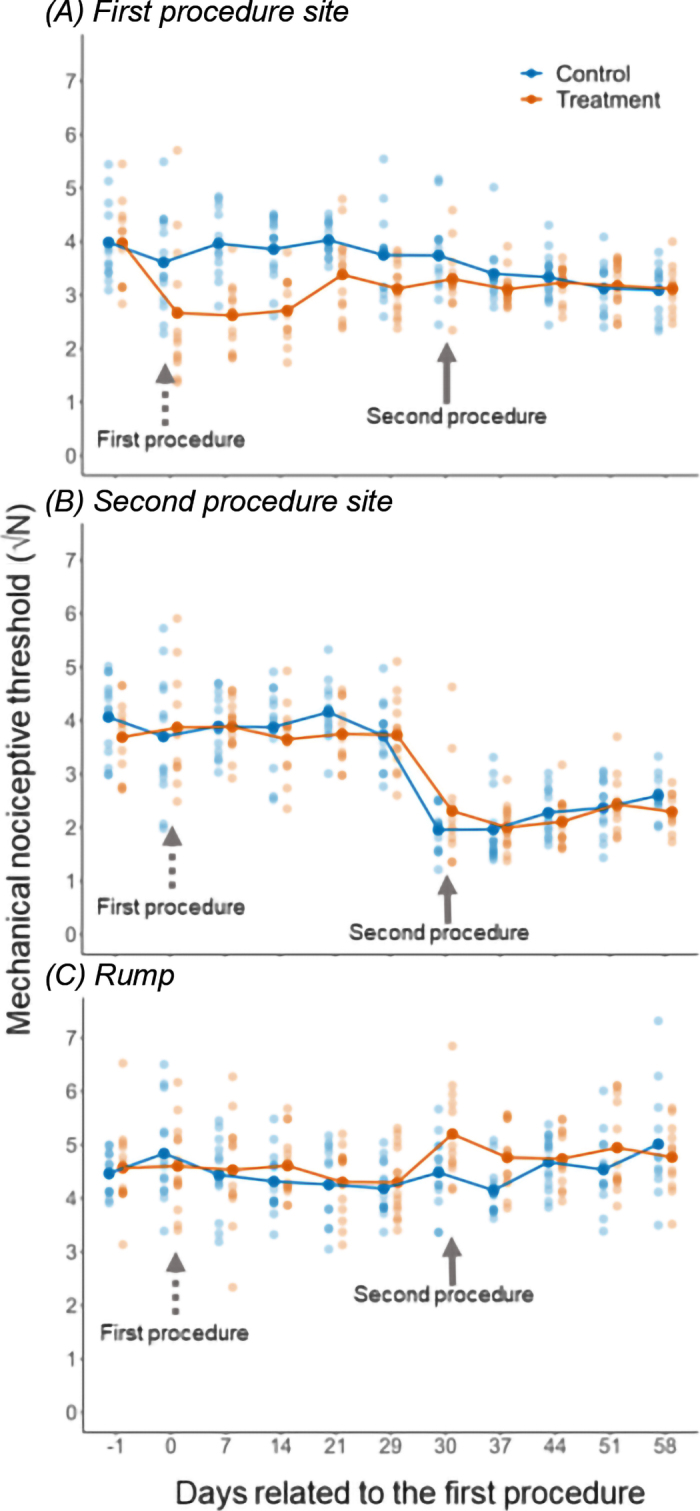
Mechanical nociceptive threshold measured at (A) the horn bud subjected to the first procedure (either sham disbudded for control calves, or caustic paste disbudded for treatment calves); (B) the horn bud subjected to the second procedure (hot-iron disbudding for all calves); and (C) the rump. Day −1 shows the average of the measurements taken on d −2 and −1 before the first procedure. Days 7, 14, 21, 29, 38, 45, 52, and 59 are the average of the 2 weekly values. The dashed arrow at d 0 and solid arrow at d 30 indicate the measurements taken 5 h after the first and the second procedure, respectively. Values are square-root transformed, and lower values indicate higher pain sensitivity. Semi-transparent dots show values from individual calves, and solid dots show treatment means.

We found no evidence of differences between treatments in the number of pain behaviors 5 h after either the first or second procedure. Across both treatments, calves averaged 6.54 ± 0.88 behaviors/5 h after the first procedure and 5.30 ± 0.95 behaviors/5 h after the second procedure.

Contrary to our expectations, a previous painful experience did not lead to a decrease in the nociceptive threshold at the rump and the second procedure site. Unexpectedly, we discovered an interaction, likely driven by treatment differences at the rump site. Interestingly, the nociceptive threshold tended to increase following the second procedure in treatment calves. These findings provide preliminary evidence of hypoalgesia in cattle associated with exposure to a previous painful event. Hypoalgesia induced by early painful experiences has been described in humans. Children, 9 to 14 yr of age, treated for at least 3 d as an infant in a neonatal intensive care unit (**NICU**), where repeated painful procedures were performed, showed a higher thermal nociceptive threshold compared with children who had not been in the NICU (Hermann et al., 2006). An increase in the number of painful procedures during the NICU was also reported to result in a lower response to sensory stimuli (i.e., immersion in cold water; Vederhus et al., 2012).

Contrary to our hypothesis, we did not find evidence of a lower pain threshold at the injury site following the second pain procedure in calves who had previously experienced pain. Previous work has shown that early-life painful experiences can amplify later painful experiences in humans and other animals. In sheep, ewes that experienced tail docking during the first week of life showed more pain-related behaviors during parturition than did control animals (Clark et al., 2014). In rats, a lower pain threshold was observed after a paw incision in individuals that had undergone a previous incision in the contralateral paw 14 d before (Walker et al., 2009). In humans, children who suffered severe or moderate burns during their first 2 yr of life showed a lower nociceptive thermal thresholds when tested at 9 to 16 yr of age (Wollgarten-Hadamek et al., 2009).

Adcock (2021) argued that the age at which the injury occurred and the age when pain sensitivity was assessed may affect the results obtained. For example, lambs castrated on their first day of life showed more pain-related behaviors after being tail docked approximately 30 d later compared with lambs castrated at 10 d of life (McCracken et al., 2010). In a study investigating the effect of different ages of hot iron disbudding on systemic pain thresholds, Adcock and Tucker (2018) found that calves disbudded at 4 d of life had a lower MNT in the rump area than calves disbudded at 40 d. Calves disbudded at 3 or 35 d of age also showed a higher heart rate responses to vaccination at 11 mo of age than calves disbudded at 56 d (Adcock and Tucker, 2020). In rats, a plantar incision at 3 and 6 d of life resulted in hyperalgesia following a second incision 14 d later; however, hyperalgesia was not observed when the first incision was made at 10, 21, or 40 d of life (Walker et al., 2009). It has also been speculated that in calves the period before 35 d of age may be particularly sensitive to pain amplification later in life (Adcock and Tucker, 2020); based on this work we chose to perform the first painful procedure when calves were ~10 d old.

One of the potential reasons for the differences in findings between the current study and this earlier work (Adcock and Tucker, 2020) is the shorter time interval between the 2 procedures adopted in our study. We used a 30-d gap between procedures, but both took place within the first 60 d of life. Our results may also reflect the efficacy of the multimodal pain management protocol applied to all calves in our study. Multimodal pain control is reportedly effective in mitigating pain induced by both hot iron and caustic paste disbudding (Stewart et al., 2009; Winder et al., 2018). The use of pain control can prevent hyperalgesia. Taddio et al. (1997) reported that human male babies circumcised at 20 d of age displayed more crying and facial expressions associated with pain when vaccinated at 4 or 6 mo, but the use of an anesthetic cream before circumcision reduced this effect.

The type of stimulus used to assess MNT may also have influenced our results. In humans, childhood burns resulted in changes in the thermal, but not mechanical nociceptive, response in 11-yr-old children (Walker et al., 2009). Although mechanical and thermal stimuli have been used to assess nociceptive threshold in cows and calves (Pinheiro Machado F° et al., 1997; Peters et al., 2015), mechanical stimuli have been widely used to assess pain threshold following disbudding. We thus opted for a mechanical stimulus in the current study, but future work should consider using more relevant stimuli.

High variability has been reported as a challenge in studies examining MNT in calves and cows (Raundal et al., 2014; Williams et al., 2020). However, few studies have investigated the underlying reasons for this variability. In an effort to minimize variation, MNT assessments were conducted by the same trained individual throughout the trial, and measurements were taken at 2 different locations per site twice a week, allowing for the use of averaged values. Despite these measures, we still found considerable variation among calves; we encourage future research to explore the sources of variability in MNT assessments. One limitation of our study is that all calves were ear tagged when they were 4 d of age. This was done to allow the use of automatic feeders and to comply with Canadian legal requirements to ensure traceability. Ear tagging is also associated with pain behaviors, including headshaking, ear scratching, tail wagging, foot stamping, and vocalization (i.e., Lomax et al., 2017; Schnaider et al., 2022), and very recent evidence indicates that the wounds can take up to 12 wk to heal (Harmon et al., 2023). This early pain experience may have affected our results.

In conclusion, we found no evidence that a painful procedure, performed at 10 d of age, results in hyperalgesia in response to hot-iron disbudding 30 d later. Indeed, our results are more consistent with hypoalgesia induced by an early painful procedure.
